# Supplementary material for: Are carbon emissions trading and green financial instruments synergistic? -Comprehensive quantitative research based on content analysis
Source: PLoS One. 2024 Mar 7;19(3):e0298601. doi: 10.1371/journal.pone.0298601 (PMC10919601; doi:10.1371/journal.pone.0298601)
Supplement: S1 Data — (ZIP) [file pone.0298601.s001.zip › Data/地级市Z_F工作报告.zip!/║╙▒▒/▓╫╓▌╩╨/└┤╫╘╣1⁄2╓┌║┼í╛┬φ┐╦╩2╛▌═°í┐_2016.docx]

各位代表：

　　现在，我代表沧州市人民政府向大会作工作报告，请各位代表连同《沧州市国民经济和社会发展第十三个五年规划纲要（草案）》一并审议，并请市政协委员和列席会议的同志提出意见。

一、“十二五”时期经济社会发展回顾

过去的五年，是我市改革发展进程中不平凡的五年。面对复杂多变的宏观环境和艰巨繁重的改革发展任务，全市上下在省委、省政府和市委的坚强领导下，坚持以“五城建设”为统领，牢牢把握稳中求进工作总基调，主动适应经济发展新常态，顽强拼搏，合力攻坚，顺利完成“十二五”规划任务目标，开创了又好又快发展的新局面，沧州站在了新的更高起点上。

综合经济实力晋级升位。围绕打造全省率先发展增长极目标，我们牢牢把握发展第一要务，壮大经济总量，提升发展质量，成功实现弯道超车、进位突破，综合实力跻身全省第一梯队。“十二五”期间，全市生产总值成功突破3000亿元大关，由2010年的2203亿元增加到2015年的3240.6亿元，年均增长9.5%，经济总量从全省第四跃居第三；全部财政收入连续突破300亿元、400亿元，达到446.7亿元，年均增长10.5%；一般公共预算收入连续突破100亿元、200亿元，达到210.9亿元，实现翻番，年均增长18.2%。规模以上工业增加值达到1257亿元，年均增长11.7%；固定资产投资3103亿

元，年均增长21.4%；社会消费品零售总额1104亿元，年均增长14%。

沿海开发建设提速推进。围绕发挥沿海区域龙头带动作用，我们建港口、搭平台、优环境、聚产业，渤海新区的影响力日益扩大。黄骅港口岸正式开放，综合保税区加快建设，20万吨级航道、20万吨级矿石码头、邯黄铁路同步建成投用。2015年，完成港口吞吐量1.67亿吨、集装箱突破50万标箱，跻身中国20大港口之列。中重高端冷轧薄板、神华机车检修、正元化肥等263个亿元以上重点项目竣工投产，世原汽车零部件、北交大综合研发实验基地等项目顺利推进。“港、产、城”协调发展格局初步形成，产业聚集能力和龙头带动作用持续增强。

重点项目建设势头强劲。围绕发挥投资拉动作用，我们启动实施重点项目“4+2”观摩考评机制和领导分包责任制，一批填补沧州产业空白的重大战略支撑项目取得突破性进展，“聚集”和“裂变”效应加速显现。五年来，全市共实施亿元以上竣工和在建项目1278个，累计完成投资6238亿元，其中，超30亿元项目34个，超50亿元18个，超100亿元8个。华北石化千万吨炼油全面开工建设；北京现代沧州工厂去年4月3日正式开工，主体已经竣工，10月份可以实现量产；北汽黄骅60万辆微车一期实现当年引进、当年投产；北京生物医药产业园签约项目48个，10家企业入驻开工；总投资1200亿元的海兴核电项目扎实推进，一期投资400亿元的核燃料产业园项目开工建设。沧州项目建设体量大、质量好、后劲足，走在了全省前列。

结构调整取得重大进展。围绕转型升级这条主线，我们改造提升传统产业，培育壮大战略新兴产业，大力发展现代服务业和现代农业。2015年上榜“全国十大质量魅力城市”，连续三年入围福布斯中国商业百强城市。三次产业结构由2010年的11.5累50.7累37.8调整为9.9累49.5累40.6。全市增加规模以上工业企业928家，达到2380家，全省第二；科技型中小企业5215家，全省第一。以汽车制造、生物医药、激光装备研发制造与应用为代表的战略性新兴产业异军突起，成为经济发展的新亮点。阿里巴巴沧州产业带上线运营，传化物流智能公路港项目顺利启动，规上服务业企业由2010年的59家猛增到370家，增长5.3倍。粮食总产完成88.9亿斤，建成青县、中捷、孟村三个省级现代农业园区，“渤海粮仓”农业科技示范园晋升为国家级园区。温氏畜牧养殖、中捷犇放建成投产，省级以上农业产业化龙头企业达到59家、农民专业合作社1.04万户，产业化经营率达到62%。

城乡面貌发生可喜变化。围绕完善城市功能、提升品位形象，我们高起点规划、高标准建设，一批重大基础设施项目相继建成投用，综合承载能力显著增强。沿海高速建成通车，环城高速成功合围，京沪高速冀鲁界工程加快推进，开通京沪高铁沧州到北京首发班列和朔黄铁路沧州到黄骅港客运专线。南水北调中线保沧干渠、石津干渠建成通水，200万人喝上长江水。城市建设全面升级，中心城区黄河路快车道、维明路立交桥建成通车，打通了东西向交通大动脉；昊天热力投产运行，改变了东部城区没有集中供热的历史；沧州体育场、规划馆、博物馆、图书馆、游泳馆、高铁西站、名人植物园等一批标志性建筑投入使用。县城建设全面提速，功能日益完善，7个县（市）获省级人居环境奖，12个县（市）成功创建省级园林县城，中心城区和肃宁县被评为国家园林城市（县城）。农村面貌改造提升行动扎实推进，建成美丽乡村1014个。全市常住人口城镇化率突破50%，城市带动农村、城乡一体发展格局初步形成。

改革开放迈出新的步伐。围绕进一步释放和增强发展活力，我们持续深化改革、扩大开放，不断增创发展新优势，拓展发展新空间。取消全部行政监管和非行政许可审批事项，市本级审批事项由449项精简到175项。“四个清单”、“三级平台”、“两个代办”制度全面建立，政务服务中心、公共资源交易中心、企业“110”和金融服务中心规范运转。市属国有集体企业改革稳步推进，市、县两级政府机构改革全面完成。在全省率先推行“六个零”企业登记和“三证合一、一照一码”制度，市场主体总数达到26.8万户，是2010年的3.3倍。众创空间、创客经济方兴未艾，大众创业、万众创新氛围空前浓厚。成功引进华夏、浦发等19家银行金融机构；全市存贷比达到55.7%，比2010年提高11.6个百分点；新增上市企业92家，达到104家，连续两年被省政府授予“上市贡献奖”。对接京津成果显著，合作项目达到955个，总投资2853亿元。沧州中心医院、中西医结合医院、市人民医院分别与北京301、天津肿瘤、北大肿瘤医院实现全面合作，北交大海滨学院、北航教育园等多所高等院校落成招生。成功举办首届冀商大会和越南、印度经贸合作推介会等招商活动，与澳大利亚维拉德、德国伟博思通等一批世界500强及跨国公司实现战略合作。中捷中欧产业园被省政府确定为河北面向中东欧开放合作的重要平台。全市累计利用外资18.88亿美元，完成外贸进出口130亿美元，境外投资5.33亿元，分别为“十一五”时期的2.2倍、1.8倍和4.3倍。

生态环境质量持续改善。围绕建成碧水蓝天、秀美宜居的生态环境，我们以壮士断腕的决心狠抓污染治理，努力让全市人民都能“深呼吸”。实施“压煤、降尘、控车、减排、增绿”工程，购置新能源公交车1693辆，淘汰黄标车16.1万辆，取缔全部366座实心黏土砖瓦窑，如期完成“十二五”减排目标，P M2.5平均浓度比2013年下降31.4%。水环境质量进一步改善，11条重点河流化学需氧量平均浓度比2010年下降32.5%，集中式饮用水源地水质达标率稳定保持100%，形成地下水压采能力1.3亿立方米。绿色行动三年计划扎实推进，完成造林面积120万亩，森林覆盖率达到27.5%。深入开展“利剑斩污”专项行动，查处环境污染刑事案件132起，抓获犯罪嫌疑人143名，有效震慑了环境违法行为。

各项社会事业全面进步。围绕不断提高人民生活水平，我们坚持政策向民生倾斜、服务向民生倾斜，投入财政资金1405亿元，办成了一批关系群众切身利益的好事、实事，成功入围2015中国民生发展百强城市。城乡居民可支配收入分别达到26350元和10291元，比2010年增长63.5%和86.2%，城镇累计新增就业29万人。社会保障体系不断完善，城镇和农村低保标准分别比2010年提高64%和100%，新农合和城镇居民医疗保险财政补助标准比2010年增长3.2倍，城乡居民社会养老保险合并实施走在全国前列，农村互助幸福院、城市社区养老服务站覆盖率分别达到65%和75%。基层医疗卫生服务能力不断提升，中医药事业得到进一步发展。开工建设保障性住房14.5万套，城镇低收入住房困难家庭实现应保尽保。扶贫攻坚成效明显，41万贫困人口稳定脱贫。素质教育深入实施，办学条件明显改善，彻底消除D级危房，生均公用经费大幅提高，实现高校全额拨款，河北工专成功升本，新一中建成投用。节会赛事精彩圆满，成功举办第十四届省运会和第十五届中国吴桥国际杂技艺术节沧州主会场。文化艺术蓬勃发展，荣获“中国工笔画之城”称号，成为中国壁画学会创作研习基地。“好人之城”温暖沧州，158名沧州好人荣登“中国好人榜”。平安沧州建设扎实推进，食品药品安全监管力度加大，安全生产形势持续好转，社会大局保持和谐稳定。国防动员和双拥共建深入开展，军政军民团结更加巩固，连续三届获得“全国双拥模范城”称号。民族宗教、外事侨务、人民防空、气象地震、史志档案、妇女儿童、老龄、残疾人等各项事业都取得了新成绩。

刚刚过去的2015年，面对不断加大的经济下行压力，我们冷静应对，趋利避害，保工业正常运转，促经济健康运行，巩固了稳中有进的良好态势，实现了又好又快发展；面对日趋激烈的区域竞争，我们主动出击，上下联动，宣传独特优势，内引外联招商，进一步扩大了沧州知名度，建成了一批引人注目、提振士气的大项目；面对风险和挑战，我们勇于担当，妥善处置，化解利益纠纷，调处矛盾冲突，全力维护了和谐稳定的社会大局，营造了齐心协力稳增长、真抓实干促发展的大气候。全市生产总值增长7.7%，全省第二；一般公共预算收入增长11.2%，全省第二；固定资产投资增长13.7%，全省第三；规模以上工业增加值增长6.9%，全省第一；实际利用外资增长38%，全省第一；社会消费品零售总额增长9.6%，全省第二；城乡居民人均可支配收入增长9%，城镇新增就业5.5万人、登记失业率控制在4.5%以内。2015年主要目标任务的完成，标志着“十二五”规划圆满收官。在推进经济社会发展的同时，我们更加重视加强政府自身建设，认真践行“三严三实”，严格落实“八项规定”，深入开展解放思想大讨论活动，政府工作效率和执行力得到不断提升。全年办理人大代表建议189件、政协提案324件，按时办复率100%。

各位代表！五年的不懈努力，为沧州未来发展奠定了坚实基础；五年的探索实践，为今后工作积累了宝贵经验。我们深刻体会到，思路决定出路，态度决定高度，适应新常态，引领新发展，必须解放思想，对标先进，跳出沧州看沧州，以更宽的视野、更高的标准，谋划沧州发展，推进各项工作；必须抢抓机遇，把握大势，围绕京津冀协同发展大战略，以承接产业转移为突破口，主动出击，精准发力，在深化对接合作中壮大自身实力，真正使机遇优势转化为发展优势；必须坚定信心，保持定力，牢牢扭住发展第一要务，困难面前不动摇，压力当头不松劲，始终保持一股唯旗誓夺、奋勇争先的精气神；必须毫不动摇，狠抓项目，把项目建设作为统领经济社会发展的总抓手，以项目扩总量、提质量，以项目夯基础、增后劲，构筑跨越发展的强劲支撑；必须立足当前，着眼长远，围绕结构优化、动能转换，做好新老接续和改造提升文章，培育壮大沧州自己的“当家产业”，在转型发展中闯出一片新天地；必须以人为本，一心为民，把全市人民的幸福期待作为政府工作的努力方向，在真诚服务和无私奉献中，凝聚推进发展的强大合力、激发干事创业的巨大热情。

各位代表！过去的五年，是全市上下同心同德、砥砺奋进，沧州实现跨越发展的五年，也是人民群众得到更多实惠的五年。五年发展的成就，得益于省委、省政府和市委的正确领导，得益于市人大、市政协和社会各界的鼎力支持，凝聚着全市广大干部群众的智慧和汗水。在此，我代表沧州市人民政府，向人大代表、政协委员，向各民主党派、工商联、人民团体和各界人士，向驻沧部队、武警官兵和政法干警，向所有关心、支持沧州发展的同志们、朋友们，致以崇高的敬意和衷心的感谢！

回顾五年来的工作，我们也清醒地看到经济社会发展中的困难和问题。主要是：发展的质量和效益还不够高，与打造增长极的目标要求还有不小差距；产业转型升级任务重，推进新动能成长和传统动能提升，需要付出更大努力；建成全面小康社会还有一些短板，发展的整体性、协调性有待进一步增强；政府职能转变有待深化，个别公务人员服务意识不强，工作缺乏激情和担当精神。对此，我们一定高度重视、认真研究解决。

二、“十三五”时期的目标和任务

“十三五”时期，是全面建成小康社会的决胜阶段。站在新的历史起点审视沧州，京津冀协同发展、“一带一路”、环渤海地区合作发展等重大战略机遇叠加，沿海、区位、交通、土地、产业等综合优势凸显，政策拉动效应、要素聚集效应相互作用，正加速转化为新的巨大能量。沧州经济持续健康快速发展，在河北的战略地位显著提升，为今后发展奠定了坚实基础、赢得了更多主动，在全省经济爬坡过坎的特殊时期，我们更有条件率先突破、直道超越，抢占发展的制高点。全市人民勤劳朴实、干部勤勉敬业、企业家队伍勇于开拓，蕴藏着无限的创新创业激情，这是沧州增比进位、跨越赶超的重要推动力量。总之，未来五年将是沧州发展历史上重大机遇最为集中的时期，是沧州各种优势和潜力最能得到有效释放的时期，是沧州实现又好又快发展最为宝贵和有利的时期，我们坚信，“十三五”将会呈现稳步开局、逐年加速的发展态势。

机遇承载使命，责任考验担当。实现人民群众对美好生活的向往，是我们奋力前行的强大动力；确保全市760万人民共同迈进小康社会，是我们责无旁贷的重要使命。我们必须倍加珍惜机遇，用足用好机遇，激情工作，勇往直前，奋力开创沧州经济社会发展新局面。

“十三五”时期全市经济社会发展的指导思想是：全面贯彻党的十八大和十八届三中、四中、五中全会精神，深入贯彻习近平总书记系列重要讲话精神，牢固树立创新、协调、绿色、开放、共享发展理念，坚持率先、转型发展要求，坚守发展、生态、民生三条底线，把握又好又快发展主基调，以提高发展质量和效益为中心，着力加强供给侧结构性改革，全面推进“五城建设”，稳增长、调结构、促改革、补短板、惠民生，确保如期全面建成小康社会，努力把沧州打造成为环渤海地区重要的沿海开放城市。

统筹考虑贯彻五大发展理念和我市经济社会发展需要，“十三五”时期主要指标设定为33项，其中约束性指标14项、预期性指标19项。总体目标是“三个率先、两个翻番、一个全面建成”。即：经济增长在全省率先、质量效益在全省率先、生态环境在全省率先；生产总值比2010年翻一番以上，城乡居民人均可支配收入比2010年翻一番以上；到2020年如期全面建成小康社会。

为实现上述目标，今后五年将重点推进七大战略任务：

（一）坚定不移推进率先发展。率先是加快发展的责任担当。必须自我加压，提升标杆，争先进位，奋力赶超。推进经济增长率先。对标沿海发达地区，明确目标，细化举措，加快发展，确保经济增长高于全省平均水平，确保综合实力稳居全省第一梯队，确保不断缩小与先进地区差距。到2020年，地区生产总值突破5200亿元，年均增长9%。推进质量效益率先。以创建“全国质量强市示范城市”为抓手，全面提高产品、工程、服务、环境四大领域质量水平。更加注重经济运行质量，确保一般公共预算收入占生产总值比重逐年提高，到2020年总量达到355亿元，年均增长11%以上。推进生态环境率先。既要金山银山，又要绿水青山，力争主要污染物排放量全省降低最快、空气达标天数全省增加最多、综合环境质量全省改善最明显，确保环境质量全国排名逐年提升，P M2.5浓度较2013年下降40%。

（二）坚定不移推进创新发展。创新是引领发展的第一动力。必须加快实施创新驱动战略，推进“创新之城”建设，让创新成为推动经济社会发展的源头活水。大力推动科技创新。突出企业创新主体地位，鼓励支持企业加大研发投入。推进科技型中小企业裂变式增长，到2020年突破10000家。加快创新孵化载体建设，大力发展众创、众包、众扶、众筹等新模式，使沧州大地创新创业竞相迸发、充满活力。积极开展金融创新。用活金融工具，创新金融产品，发展普惠金融，构建多层次、多元化的金融服务体系。引导金融机构持续扩大信贷投放，2020年金融机构存贷比达到或超过全省平均水平。培育发展基金产业，拓展直接融资渠道，三年内设立100只基金，上市企业达到200家以上。深入实施管理创新。推进政府购买公共服务，增加公共服务有效供给。加快“智慧城市”建设，建立大数据中心，形成全新的城市运营管理和服务体系。全面推进人才创新。实施人才优先发展战略，重点引进一批能够突破关键技术、引领高新技术产业发展的高层次人才，带项目、带技术、带团队来沧州创新创业。

（三）坚定不移推进转型发展。转型是经济建设的主攻方向。必须更加注重供给侧结构性改革，强化质量效益导向，推动产业产品向中高端迈进，打造“产业之城”。大力发展先进制造业。改造提升“五大”传统产业，推动石化产业精细化、管道装备高端化、机械制造智能化、食品和纺织品牌化，由“沧州制造”向“沧州创造”转变、“沧州产品”向“沧州品牌”转变。培育壮大汽车制造、生物医药、清洁能源、通用航空、节能环保、激光研发应用、再制造等“七大”新兴产业，依托龙头，加快配套，促进聚集，膨胀规模，成为全市经济增长的重要支撑力量。大力发展现代服务业。积极发展现代物流、金融保险、健康养老、商务、文化、旅游等九大产业，推动生产性服务业专业化、生活性服务业便利化、公共服务业均等化，把服务业打造成为经济增长“稳定器”和结构调整“加速器”，确保到2020年服务业增加值占生产总值比重达到50%。大力发展现代农业。加快转变农业发展方式，坚持用现代发展理念引领农业，用现代物质条件装备农业，用现代科技改造农业，用现代经营模式推进农业。加强现代农业园区建设，推动粮经饲统筹、农林牧渔结合、种养加一体、一二三次产业融合发展。

（四）坚定不移推进协调发展。协调是持续健康发展的内在要求。必须在协调推进中增创发展优势，在加强薄弱领域中增强发展后劲。统筹推进“三大经济板块”。推动沿海经济跨越发展，促进要素聚集、项目聚集、产业聚集，力争主要经济指标五年翻一番。推动中心城区突破提升，做大做强城市经济，不断提高城市首位度。推动县域经济增比进位，支持各县（市）培育壮大特色优势产业，打造一批营业收入超百亿元的产业集群，力争更多县（市）进入全国百强县。统筹推进城乡一体发展。坚持市县乡村“四位一体”，优化空间布局，促进人口聚集，构建定位清晰、特色鲜明、功能完善、生态宜居的现代城镇体系，到2020年常住人口城镇化率达到60%。围绕沧州市区和黄骅新城“双城”建设，加快发展大沧州都市区。按照“小县大县城”的思路，强化规划引领、产城教融合，推动县城建设上水平、上品位，培育一批中小城市和特色小城镇。按照“四美五改”要求，实施民居改造、污水治理等专项行动，到2020年具备条件的农村全部建成美丽乡村。统筹推进重大基础设施建设。完善港口、交通、水利、电力、信息网络等现代基础设施，全面提高支撑保障能力。加快推动邯港、曲港、任德高速公路前期工作；启动实施307、104国道绕城改线，205国道改造提升和青沧快速路工程；突出抓好石衡沧港城际铁路建设，力促年内开工、早日投用，结束沧州到省会没有直通铁路的历史。统筹推进物质文明和精神文明协调发展。围绕打造“文化之城”“好人之城”，大力加强精神文明建设，积极创建全国文明城市，推进全市人民人文素养和道德水平全面提高，构建富有美誉度、吸引力和竞争力的良好人文环境。

（五）坚定不移推进绿色发展。绿色是永续发展的重要基础。必须更加注重人与自然和谐共生，建设美好家园，打造“生态之城”。着力提升生态环境质量。采取强有力的防治措施，深入推进大气、水、土壤污染防治行动，坚决打赢治霾攻坚战。加大河流湿地保护修复力度，加快地下水超采综合治理，“十三五”末基本实现地下水采补平衡。持续开展植树造林行动，全市森林覆盖率达到33%。推进高效循环利用资源。强化约束性指标管理，实行能源和水资源消耗、建设用地等总量和强度“双控制”，坚决淘汰落后产能，大力发展循环经济，推进产业生态化、生态产业化。建立环境污染追责机制。严格落实党政同责、一岗双责、失职追责的领导干部责任制，完善属地管理、企业主体责任追究制度，严厉打击环境违法行为，严守生态保护红线。

（六）坚定不移推进开放发展。开放是繁荣发展的必由之路。必须用更大的决心和勇气深化改革、扩大开放，为经济社会发展注入新动力。全面深化行政审批、财政管理、投融资体制、国有企业等各领域改革，更加注重发挥市场在资源配置中的决定性作用。毫不动摇地鼓励支持和引导非公有制经济发展，保证民营经济主体公平参与市场竞争。加强与京津的对接合作，让更多转移项目在沧州落地生根、开花结果。推动建立津冀协同发展示范区，争取渤海新区成为天津自贸区政策延伸区。充分发挥黄骅综合大港欧亚大陆“桥头堡”的纽带作用，更加主动融入“一带一路”国家战略，加强与日韩、欧美等发达国家和新兴市场经济体的交流合作，全力构筑对外开放新格局。

（七）坚定不移推进共享发展。共享是推动发展的根本目的。必须坚持以人为本，优化财政支出结构，确保民生支出稳步增长。实施更加积极的就业政策，保持就业形势总体稳定。完善社会保障体系，提高保障水平，扩大保障范围。办好人民满意教育，促进教育公平。加快健康沧州建设，不断满足群众医疗服务需求。实施脱贫攻坚行动，确保率先完成脱贫目标。精心培育武术、杂技两大节庆品牌，积极争取中国吴桥国际杂技艺术节回归沧州。深化平安沧州建设，切实维护社会和谐稳定大局。

各位代表，“十三五”发展蓝图已经绘就，目标鼓舞人心。只要我们树立必胜信念、继续埋头苦干，万众一心、奋力拼搏，就一定能够实现全面建成小康社会的奋斗目标，就一定能够谱写沧州率先发展的新篇章！

三、2016年经济社会发展的重点工作

今年是全面建成小康社会决胜阶段的开局之年，也是推进结构性改革的攻坚之年，做好今年的政府工作至关重要。我们要认真贯彻落实党的十八届五中全会、中央和省经济工作会议、市委八届七次全会精神，把握中央推进供给侧结构性改革的重要机遇，落实“三去一降一补”五大任务，把淘汰落后产能、解决中低端产品多、终端产品少的问题作为重要突破口，把帮助企业降成本、提效益作为关键着力点，把提高全要素生产率、扩大有效供给作为核心平衡点，打好存量化解歼灭战和扩大有效供给攻坚战，加快发展动能转换和质量效益提升，促进经济社会又好又快发展。今年的主要预期目标是：地区生产总值增长8%，规模以上工业增加值增长8%，一般公共预算收入增长8%，全社会固定资产投资增长18%以上，直接利用外资增长20%以上，社会消费品零售总额增长9%，城乡居民人均可支配收入增长9%，常住人口城镇化率提高2个百分点，P M2.5浓度下降6%以上，其他节能减排指标控制在省定范围内。在实际工作中，我们将向着更高的目标努力，使经济社会发展得更好更快一些。重点在九个方面实施攻坚突破。

（一）在推进沿海经济率先发展上攻坚突破。着眼完善功能、提升能力，加快建设大港口，发展大产业，再掀沿海开发建设新热潮。一是加快提升港口运营能力。瞄准建成现代化国际一流综合大港目标，抓好系列码头群建设，力争化工、原油、滚装等12个码头顺利开工。进一步完善口岸查验设施，提升通关运营能力，力争全年吞吐量达到2亿吨，集装箱100万标箱，确保黄骅港综合保税区申报成功、封关运行。二是加快提升产业聚集能力。加快“九大功能园区”建设，精心打造汽车、石化、生物医药、冶金装备、现代物流等五个千亿元园区。全力抓好中国轨道交通综合实验基地、北交大综合研发实验基地、北汽集团汽车试验基地和汽车零部件再制造基地等重点项目前期工作，力促海伟丙烷加氢、中亚化工装备等项目开工建设，确保金牛化工40万吨P V C、星宇汽车零部件等项目竣工投产。三是加快提升港腹联动能力。采取合作共建等方式加快“内陆港”、物流基地建设，进一步扩大以集装箱为重点的海铁联运、多式联运。加快开通“黄-新-欧”海铁联运班列，以及到韩国的滚装班轮和到东南亚国家的外贸航线，推动黄骅港由现代集疏大港向国际贸易大港转变。四是加快提升港城承载能力。进一步优化“一市四区”空间布局，抓好道路、管网、通讯等基础设施建设，完善住宿餐饮、教育医疗、娱乐休闲等配套设施，为入区企业提供一流服务环境。

（二）在重点项目建设上攻坚突破。着眼充分利用中央政策调整“窗口期”的难得机遇，加快产业支撑和重大基础设施项目建设步伐，努力打造新的增长点。一是围绕结构调整抓项目。按照“一二三产并举、大中小项目齐抓”的思路，全力抓好三次产业项目的谋划与实施，以项目结构的优化升级，促进产业结构的转型发展。对符合产业政策、适应消费需求、高质高端高新的项目，给予重点支持。确保全年新开工亿元以上重点项目200个，竣工100个，完成投资1900亿元。二是围绕战略支撑抓项目。坚持实行领导分包责任制和观摩考核机制，着眼发挥重大项目“聚集”和“裂变”效应，牢牢抓住总投资5814亿元的36个战略支撑项目，确保北京现代沧州工厂竣工投产，河北京丰电力高压传输设备开工建设，华北石化千万吨炼油、核燃料产业园快速推进；加快80万吨P X、120万吨P T A、100万吨芳烃等项目前期工作，力促海兴核电取得突破性进展。三是围绕产业集群抓项目。依托特色抓聚集，依托产业链抓聚集，成板块、链条式引进配套项目，加快推进泊头数控机床、东光包装机械、肃宁裘皮、盐山和孟村管道装备等重点园区建设，力争沧州开发区主营业务收入超350亿元、黄骅和青县开发区超200亿元。四是围绕争跑政策资金抓项目。研究掌握国家和省稳增长、调结构的系列政策措施，精心谋划包装项目，强化对口跑办，以项目争政策、引资金，争取政策红利最大化。重点运用好政府和社会资本合作（P P P）模式，围绕港口、道路和城市基础设施建设，加快洽谈对接，加大资金引进力度，力争全年运作100个成熟项目。

（三）在产业转型升级上攻坚突破。着眼把握供给侧结构性改革的有利契机，做好“加减乘除”四则运算，加快培育经济增长的新动能。一是加大传统产业改造力度，做强存量。开展全国质量强市示范城市创建活动，深入实施区域产品质量提升工程，叫响“沧州质量”品牌。实施“三个一百”工程，重点抓好100家“两化”融合企业、100个技术改造项目、100个新产品开发，推动传统行业产品升级、链条延伸、效益提升。搭建“个转企、小升规、规改股、股上市”成长阶梯，年内新增规模企业150家，每个县（市、区）至少新增1家上市企业。加快“僵尸企业”资产处置，通过兼并重组、优胜劣汰，倒逼低效无效产能退出，将资源要素向优质企业倾斜。二是大力发展战略新兴产业，做大增量。实施七大新兴产业发展“助力工程”，在项目引进、要素保障和市场开拓等方面给予重点扶持，确保新兴产业增加值增长10%以上。重点抓好汽车产业园、激光产业园、生物医药园、通用航空城和再制造产业基地等园区建设，支持激光加工国家工程研究中心京津冀区域中心申报省级工程研究中心和省级技术研发中心、河间再制造申报国家级示范基地。三是推动服务业提速发展，增加体量。落实《关于加快推进现代服务业发展的实施意见》，实施九大行业发展三年行动计划。加快发展现代物流业，做强渤海新区、肃宁、沧东3个省级物流园区，抓好传化物流智能公路港、好日子物流中心等项目。加快发展电子商务，推进阿里巴巴产业带、沧州国际电子商务产业园、腾讯“智慧运河”等项目建设，打造“沧州电商品牌”。加快发展健康养老业，支持颐和乐园老年公寓、献县颐康休闲养老园、任丘“医康养”生态养老基地建设。加快发展文化旅游业，依托武术、杂技等历史文化资源，积极推进吴桥杂技文化旅游产业园、任丘白洋淀生态旅游度假区等重点项目建设。规范房地产企业经营行为，合理引导住房消费预期，促进房地产业平稳健康发展。四是促进农业增产增效，提升质量。加强粮食生产核心区建设，扎实推进“渤海粮仓”科技示范工程，推广示范面积300万亩，增粮6亿斤。做优做强畜牧、蔬菜、果品三大优势产业，抓好孟村大成、青县周黑鸭、澳洲肉牛加工等龙头项目，打造京津农副产品生产供应基地。把农业园区建设作为发展现代农业的突破口，推动涉农资金、项目打捆集中向园区投放，力争建成省级现代农业园区7个、农产品出口示范基地5个，农业产业化经营率达到64%。

（四）在城乡统筹协调发展上攻坚突破。着眼京津冀区域打造世界级城市群、全省建设新型城镇化和城乡统筹示范区的大战略，全面提升城市发展水平，补齐沧州新型城镇化建设的短板。一是高水平建设中心城区。贯彻落实中央和省城市工作会议精神，坚持空间、规模、产业相协调，规划、建设、管理相衔接，以建设全国海绵城市、森林城市、生态园林城市为抓手，实施总投资410亿元的重点城建工程，推动城市内涵式发展。（1）做强城市经济。按照“以产兴城、以城带产、产城融合”的思路，大力发展高新技术产业和高端服务业，不断提高中心城区对全市发展的带动力和贡献率。依托“三区两园”，抓好北京二七轨道交通、海纳川汽车配件、中国国际机器人等重点项目建设。实施城市商圈和楼宇经济培育工程，重点打造颐和广场、荣盛国际、千童商业广场等5大商圈，抓好21个企业总部建设，建成一批税收超千万元的商务楼、写字楼。（2）做优城市功能。围绕让市民生活更舒适、更便捷，持续加大投入力度，新建续建饶安、海丰等27条城市道路，启动永济路高架和渤海路改造提升工程，确保九河路全线贯通，畅通东西交通大动脉；抓好青海大道综合管廊、华润运东热电等项目，新增集中供热能力110万平方米；加快青少年宫、妇女儿童活动中心建设，确保尽快投入使用。（3）做美城市环境。突出做好“水”“绿”两篇文章，让城市更有灵气、更加靓丽。抓好人民、双金、动物园等10个园林绿化项目，新增绿化面积391万平方米。围绕构建“一河五环十湖百塘”的多层次水网体系，加快推进滨河公园二期、小流津河等改造提升工程，突出抓好运河景观带改造，把大运河沿岸打造成为高品质的城市景观和文化旅游走廊。（4）做细城市管理。加快建设“智慧沧州”，全面推广数字化管理模式，实现城市管理精细化、精准化。启动智慧泊车工程，完成主城区26.7平方公里区域内公共停车场的管理移交和规范建设工作。实施黄河路、新华路、北京现代沧州工厂周边等7条道路两侧景观改造、亮化和牌匾整治，进一步提升城市形象。二是促进县城扩容提质。实施县城建设三年攻坚行动，进一步拉大县城框架，把周边3-5公里全部纳入规划范围，促进产城教融合发展；进一步完善城市功能，提升承载能力，所有县城实现集中供水供热、污水垃圾达标处理。加快发展县域经济，重点抓好27个年营业收入超50亿元的产业集群，培育一批龙头企业和区域品牌。三是加快美丽乡村建设。加大基础设施和公共服务设施投资，提升完善乡村公路，搞好农村电网改造，高标准完成492个省级重点村的建设任务。重点抓好吴桥杂技大世界省级精品片区、中心城区高速合围片区建设，力争每个县（市）建成1-2个新片区。四是提升城镇人口规模。加快区划调整，积极推进乡改镇、镇改街、村改居。深化户籍改革，实施居住证制度，建立健全“人地钱”三挂钩机制，全市常住人口城镇化率提高到52%。

（五）在激发创新创业活力上攻坚突破。着眼发挥创新创业对推动转型发展、动能转换的关键作用，搭建优质平台，营造良好环境，让沧州成为创新要素生成的“沃土”，创业人才成长的“乐园”。一是突出培育创新主体。深入实施高新技术产业三年倍增和科技型中小企业成长计划，确保年内新增科技型中小企业1000家以上、“科技小巨人”企业50家、高新技术企业30家以上。支持企业采取自办或校企共建方式，建设工程技术研究中心、产业研究院、院士工作站等17家技术创新平台。二是加快协同创新步伐。依托京南G45科技成果转化试验区、激光加工国家工程研究中心京津冀区域中心、北京科创园渤海新区产业基地和国家再制造产业示范基地，深化与京津科研院所合作，共建一批产业园区和科技成果孵化基地，打造承接京津科技资源转移、转化的集聚区。三是培育壮大金融市场。全面落实加强金融产品创新、推动实体经济快速发展的措施，力争全市金融机构存贷比达到60%以上。大力发展政府引导基金，加强与域外大型投资机构的合作，年内基金数量达到25支以上。组建交通投资公司、金融投资公司，推进农村信用社改制工作，支持“一行两投”做大做强。加强政府债务管控，完善地方金融监管体制，依法打击非法集资等违法行为，坚决守住不发生系统性和区域性金融风险底线。四是深度激发“双创”活力。把“双创”“互联网+”等融入各领域各环节，出台更具吸引力的政策措施，让创新创业遍地开花、“草根”经济枝繁叶茂。抓好北航科技园、清华启迪科技孵化器等平台载体建设，全年新增市场主体6万户以上。深入开展企业家培训活动，支持民营企业“二次创业”，加快转型升级、焕发新的活力。

（六）在深化改革开放上攻坚突破。着眼进一步扩大京津冀协同发展成果，坚持以改革促开放，以开放促改革，聚集先进要素，发展外向型经济。一是加快转变政府职能。以“放管服”为重点，深化行政审批制度改革，完善“四级平台”“两个代办”机制，变“群众奔波”为“信息跑腿”，打造法治化、便利化的营商环境。建立网上行政审批与公共服务系统，促进信息资源共建共享。加强行政审批标准化建设，抓好电子营业执照试点工作，深入推进“四个清单”和项目联合审批管理制度，全面落实“两集中、两到位”。二是推进重点领域改革。加快市属国有集体企业股份制、产权多元化改革，重点抓好30家企业改制工作。深化农村综合改革，引导规范农村土地经营权依法有序流转。抓好绩效预算管理改革，全面推进“营改增”，推进结构性减税和普遍性降费。深化开发区管理体制改革，完成市县公务用车改革，统筹推进教育、医疗、文化等领域改革。三是扩大对内对外开放。充分发挥国家部委、京津政府、央企、中关村、科研院所和高等院校等“五个对接小组”作用，吸引更多优质产业项目落户沧州。依托22个省级以上开发区，瞄准国内外500强和行业领军企业，大力实施领导定向招商、产业精准招商、园区特色招商、主题推介招商。抓好综合保税区和中欧、中韩等国别产业园建设，打造对外开放新平台。支持本地企业开展境外投资，抢占海外市场，拓展外经空间，扩大外贸出口。

（七）在生态环境建设上攻坚突破。着眼实现生态环境率先，坚决打好环境治理攻坚战，建设天蓝、地绿、水清的美丽沧州。一是持续推进大气污染防治行动。实施大气污染治理三年计划，推进燃煤治理和清洁型煤替代，取缔市区所有10蒸吨以下燃煤锅炉。加大料场、煤场、混凝土搅拌站的监管力度，确保实现标准化、规范化运营。深入开展扬尘专项整治行动，持续抓好清扫保洁、渣土车管理、“黄标车”淘汰、露天烧烤整治和小企业群治理等工作。健全完善调度考核机制，保持上下联动、齐抓共管、网格监控、追责问责的高压态势。二是持续推进水环境综合治理。落实水污染防治“国10条”和“省50条”，配合做好白洋淀综合整治工作，组织开展重污染河流环境治理攻坚、集中式水源地安全防护等专项行动。加快乡镇污水处理厂升级改造，全市所有工业园区建成污水集中处理设施。扎实开展地下水超采综合治理，确保完成省下达的任务目标。三是持续推进绿色沧州攻坚行动。大力开展绿色廊道、成方连片造林和城郊森林公园建设，构筑绿带成网、“森林围城”格局。完成造林绿化面积40万亩，全市森林覆盖率达到28.5%。

（八）在精准扶贫精准脱贫上攻坚突破。着眼落实“六个精准”要求，深入实施“六个一批”行动计划，确保7个贫困县全部脱贫摘帽。一是发展产业促进脱贫。深入实施贫困地区“一村一品”发展计划，突出抓好“牧、菜、菌”三大产业，扶持发展一批贫困人口参与度高的农业园区、龙头企业和农民专业合作社。二是完善基础设施保障脱贫。加大投入力度，加强水、电、路等基础设施建设，破除贫困地区发展瓶颈制约。贫困地区通村公路建设补助标准提高50%，将92个贫困村纳入美丽乡村建设实施范围。三是抓好教育医疗帮助脱贫。免除市内公办普通高中、中职学校、普通高校就读的贫困家庭学生学费、住宿费、教科书费，实施基本医疗保险、大病保险、医疗救助三重医疗保障，将贫困人口全部纳入重特大疾病救助范围。四是用好政策扶持脱贫。严格落实1%以上公共财政预算收入专项用于扶贫的规定，确保每一分钱都用在群众脱贫上。实施农村低保标准与扶贫标准“两线合一”，将符合条件的贫困人口全部纳入低保范围，兜住贫困地区民生底线。

（九）在保障和改善民生上攻坚突破。着眼提高民生保障和服务供给能力，持续加大民生投入，努力让发展更有温度，幸福更有质感。重点实施十大民生工程。一是就业促进工程：统筹解决好高校毕业生、城镇困难人员、退役军人就业，年内全市新增城镇就业5.5万人，失业登记率控制在4.5%以内。二是收入增长工程：全面落实机关事业单位工资改革措施，建立健全企业薪酬调查制度，多渠道增加农民收入，促进收入增长与经济发展同步。三是社保提质工程：稳步提高各项社会保险待遇水平，全面实施机关事业单位养老保险制度改革。四是住房保障工程：开工棚户区改造住房1.5万套，改造农村危房5000户。五是教育提升工程：坚持教育优先，推进各级各类教育内涵发展。深入实施“全面改薄”工程，创建标准化中学50所、一类以上幼儿园60所。大力发展中、高职贯通的现代职业教育，推行产教联盟、校企一体化办学，建设公共实习实训基地，积极做好沧州医专升本准备工作。六是医疗健康工程：深化医药卫生体制改革，推进大中型医院集团化管理，抓好12类45项基本公共卫生服务项目。进一步提高新农合参保农民受益水平，政策范围内住院费用支付比例提高到75%左右。支持发展中医药事业，建成69个乡镇卫生院和社区卫生服务中心“国医堂”。全面落实两孩政策，完善计生服务管理。七是幸福养老工程：加强特困人员供养服务机构建设，扶持发展社会办养老服务机构，社区养老服务站覆盖率达到80%，农村互助幸福院达到70%。八是文体惠民工程：精心组织好第九届中国?沧州国际武术节，扎实推进第三批国家公共文化服务体系示范区创建工作。加强城乡公共文体设施建设，完善14个县级图书馆，改造提升56个乡镇综合文化站，为200个行政村配备体育健身器材，市区各类院校操场和运动设施早晚时段全部对社会免费开放。九是饮水安全工程：打通李家岸引黄线路，完成南水北调中线水厂以上输水管道和石津干渠延长段建设。启动饮水安全巩固提升工程，让全市人民喝上干净水、400万人喝上长江水。十是平安沧州创建工程：加快推进视频监控全覆盖工程，打造立体化社会治安防控“升级版”。加强消防、交通、危爆物品、食品药品等领域安全管理和安全生产大检查，坚决杜绝重特大安全事故发生。深入开展法制宣传教育，增强全体公民的法治观念。推进群众工作中心建设，用好网上信访受理平台，畅通群众诉求表达渠道，及时化解矛盾纠纷。加强对各类风险源研判分析，有效化解各类风险隐患。严厉打击各类违法犯罪行为，营造安定和谐的社会环境。

四、全面提升政府建设水平

　　适应新常态、开启新征程，政府工作必须有新状态、新作风。我们一定要牢记全面建成小康社会的重大历史使命，按照忠诚、干净、担当、实干的要求，恪尽职守，永不懈怠，创造无愧于时代、无愧于人民的新业绩。

（一）解放思想，锐意创新。坚持把学习贯彻落实习近平总书记系列重要讲话精神，作为新形势下解放思想的根本要求，对照“八破八立”，着力解决思想观念、体制机制等方面的突出问题。牢固树立并践行创新、协调、绿色、开放、共享发展的理念，对标先进，聚焦短板，打破思维定式，突破路径依赖，创新思路方法，以思想的大解放促进经济的大发展。

（二）廉洁从政，从严治政。认真落实党风廉政建设责任制，强化“一岗双责”，扎紧制度围栏，严守廉洁底线。严格执行“八项规定”和《准则》《条例》，树立政治意识、大局意识、核心意识、看齐意识，把政治纪律和政治规矩挺在前面。加强行政监管和审计监督，坚决纠正损害群众利益的不正之风，努力实现干部清正、政府清廉、政治清明。

（三）依法行政，科学施政。加强法治政府建设，严格执行政府学法制度和重大行政决策程序，切实做到依法依规行使权力。推进政务公开，深化重点领域、重大民生项目信息公开。坚持自觉接受人大法律监督和政协民主监督，广泛听取各民主党派和社会各界人士的意见建议，让政府工作更加科学、更加民主、更加符合人民的意愿。

（四）夙兴夜寐，激情工作。把“三严三实”作为行动标尺，贯穿到方方面面，让夙兴夜寐、激情工作成为常态。坚持干字当头、快字为先，对重点项目、重大事项、重要工作，快谋划、快上手、快实施。大力弘扬敢于负责、勇于担当、狠抓落实的作风，加强跟踪督导，强化问责问效，确保各项工作高标准推进、高质量完成。

各位代表！百舸争流，破浪者领航；千帆共进，奋勇者当先。勤劳智慧、自强不息的沧州人民，在改革开放的征途上创造了不凡业绩，也一定能够在时代发展的大潮中再铸新的辉煌。让我们在省委、省政府和市委的坚强领导下，勠力同心，锐意进取，为加快沧州“五城建设”，全面建成小康社会而努力奋斗！
